# Applications of Artificial Intelligence in the Neuropsychological Assessment of Dementia: A Systematic Review

**DOI:** 10.3390/jpm14010113

**Published:** 2024-01-19

**Authors:** Isabella Veneziani, Angela Marra, Caterina Formica, Alessandro Grimaldi, Silvia Marino, Angelo Quartarone, Giuseppa Maresca

**Affiliations:** 1 Department of Nervous System and Behavioural Sciences, Psychology Section, University of Pavia, Piazza Botta, 11, 27100 Pavia, Italy; isabellamaria.veneziani01@universitadipavia.it; 2 IRCCS Centro Neurolesi “Bonino-Pulejo”, S.S. 113 Via Palermo C. da Casazza, 98124 Messina, Italy; angela.marra@irccsme.it (A.M.); alessandro.grimaldi01@universitadipavia.it (A.G.); silvia.marino@irccsme.it (S.M.); angelo.quartarone@irccsme.it (A.Q.); giusy.maresca@irccsme.it (G.M.)

**Keywords:** artificial intelligence, machine learning, mild cognitive impairment, dementia, neuropsychological assessment

## Abstract

In the context of advancing healthcare, the diagnosis and treatment of cognitive disorders, particularly Mild Cognitive Impairment (MCI) and Alzheimer’s Disease (AD), pose significant challenges. This review explores Artificial Intelligence (AI) and Machine Learning (ML) in neuropsychological assessment for the early detection and personalized treatment of MCI and AD. The review includes 37 articles that demonstrate that AI could be an useful instrument for optimizing diagnostic procedures, predicting cognitive decline, and outperforming traditional tests. Three main categories of applications are identified: (1) combining neuropsychological assessment with clinical data, (2) optimizing existing test batteries using ML techniques, and (3) employing virtual reality and games to overcome the limitations of traditional tests. Despite advancements, the review highlights a gap in developing tools that simplify the clinician’s workflow and underscores the need for explainable AI in healthcare decision making. Future studies should bridge the gap between technical performance measures and practical clinical utility to yield accurate results and facilitate clinicians’ roles. The successful integration of AI/ML in predicting dementia onset could reduce global healthcare costs and benefit aging societies.

## 1. Introduction

In an era marked by exceptional technological advances, the diagnosis and treatment of cognitive disorders have become a central frontier in health care. Among these disorders, Mild Cognitive Impairment (MCI) and Alzheimer Disease (AD) are of capital importance because of their constant impact on society.

MCI is a clinical condition that can be placed halfway between physiological and pathological aging. Patients with MCI have mild cognitive deficits (memory problems, language, thought) that are still higher than those of their age. Nevertheless, these issues tend not to interfere with their daily life activities, autonomy or social relationships [1,2]. Dementia, also known as “major neurocognitive disorder”, instead compromises cognitive, behavioral, mood and personality functions, resulting in a significant alteration of the patient’s functional state, with effects on autonomy and relationships [3]. Studies have shown that patients with mild cognitive deficits may develop dementia more frequently than healthy individuals [4]. MCI can be “amnestic” and “non-amnestic” and the two forms are characterized by the impairment of a single cognitive function (single domain) or multiple cognitive functions (multidomain) [4,5,6]. The four subtypes of MCI that emerge can evolve into one of the four main forms of dementia: AD, vascular, frontotemporal, and Lewy bodies [4,7]. In particular, the subtype amnestic/single domain is due to AD being more frequent than other subtypes of dementia [8]. Fronto-Temporal Dementia (FTD) is a neurodegenerative disorder that is characterized by a range of cognitive impairments, including deficits in executive functions, behavior, and/or language. Vascular dementia, on the other hand, is a variant caused by cerebrovascular disease, and its cognitive impairment is non-specific, as it depends on the side of brain lesions [5,6]. Additionally, non-amnestic MCI due to Lewy Bodies Dementia (LBD) is diagnosed based on the presence of attention and/or visuospatial deficits [7]. Usually, normal aging, MCI, and dementia are distinguished using clinical criteria and measurements of cognitive function, neuropsychological testing, biomarkers, or neuroimaging techniques [4]. In recent decades, with the lack of pharmacological therapies, there has been a new scientific focus on treating dementia. Specifically, for AD, the most common form of dementia in older adults, studies have suggested that modulating systemic innate immune cells could be a promising therapeutic approach [9].

MCI and AD represent increasing clinical and social health challenges worldwide, as life expectancy continues to rise globally and the incidence of these neurodegenerative conditions keeps growing. MCI incidence is, indeed, estimated to be between 5.1 and 168 cases per 1000 years/person and the prevalence rate is at 5.9% in the general population over 60, with an age group increase of 4.5% between 60 and 69, 5.8% between 70 and 79, and 7.1% between 80 and 89 years [10]. The condition of MCI may remain stable, regress into normality or evolve into dementia; the 3-year stability rate of MCI is 34%, while the remission rate is about 16% [11]. The annual conversion rate to AD is 10–15% [2,12]. Older individuals, women, ApoE4 carriers, those with fewer years of education, lower scores on the Mini–Mental State Examination (MMSE), vascular disease, and late-life depression are at the highest risk [13,14,15]. To address these challenges, we need to identify these conditions in advance, use an accurate neuropsychological assessment, and create personalized treatment plans.

In recent years, Artificial Intelligence (AI) and Machine Learning (ML) have become progressively more important in the early detection and treatment of these diseases.

AI is a broad field of computer science that creates machines, systems, or software that can perform tasks that typically require human intelligence. ML is a subfield of AI. It focuses on developing algorithms and statistical models that enable computers to improve their performance on a specific task through data analysis, without having to be explicitly programmed [16,17,18]. AI exceeds the limits of computation and analysis by excelling in the complex identification of patterns that can emerge in any data series, and can do so on a greater scale and at a greater speed than human capabilities [19]. In clinical practice, AI and ML provide unique opportunities to analyze complex networks of biological, clinical, and imaging data, enabling a better understanding of the underlying mechanisms of MCI and AD and helping healthcare professionals to obtain accurate diagnoses or identify more suitable treatment paths. Furthermore, these technologies allow the creation of advanced predictive models to identify individuals at risk of developing these conditions at an early stage, when therapies can be more effective [20]. However, a clinician’s understanding of the pure data is not—and probably will never be—replicable by computers. Collecting a treatment recommendation provided by AI and deciding whether or not it is right for the patient must always depend entirely on human decision making. As the accuracy of AI systems increases, we may begin to see a positive mutation in the role of clinicians: from being data collectors and analyzers to being interpreters and consultants for patients seeking answers about their health.

Previous studies investigating the role of AI and ML in MCI and dementia detection have generally focused on identifying biomarkers through the training of algorithms on historical data [21]. AI performance is typically discussed in studies using technical measures of accuracy, with little focus on clinical outcomes, risks, and utility. The present review aims to highlight how AI and ML can significantly contribute to the early diagnosis of and treatment optimization for MCI and AD while reducing the workload of professionals. It will examine studies that combine the principles of neuropsychology with the potential of AI and ML to identify cognitive markers, develop innovative diagnostic tools, and customize therapies based on the cognitive profiles of patients. This work would like to show the transformative potential of these technologies. These advances have the possibility to optimize the field of neuropsychological assessment, improving the quality of life for individuals affected by MCI and AD while reducing the burden on healthcare systems and caregivers.

Therefore, the objective of this study is to analyze the application of AI systems in the neuropsychological assessment of dementia.

The rest of the paper is organized as follows: Section 2 explains how the studies considered in this review were selected and what search strategies were used. In Section 3, the results and the number of studies considered are presented. Finally, Section 4 discusses the articles where AI and ML methods were applied in the neuropsychological assessment of patients with MCI and dementia.

## 2. Materials and Methods

**Figure 1:**
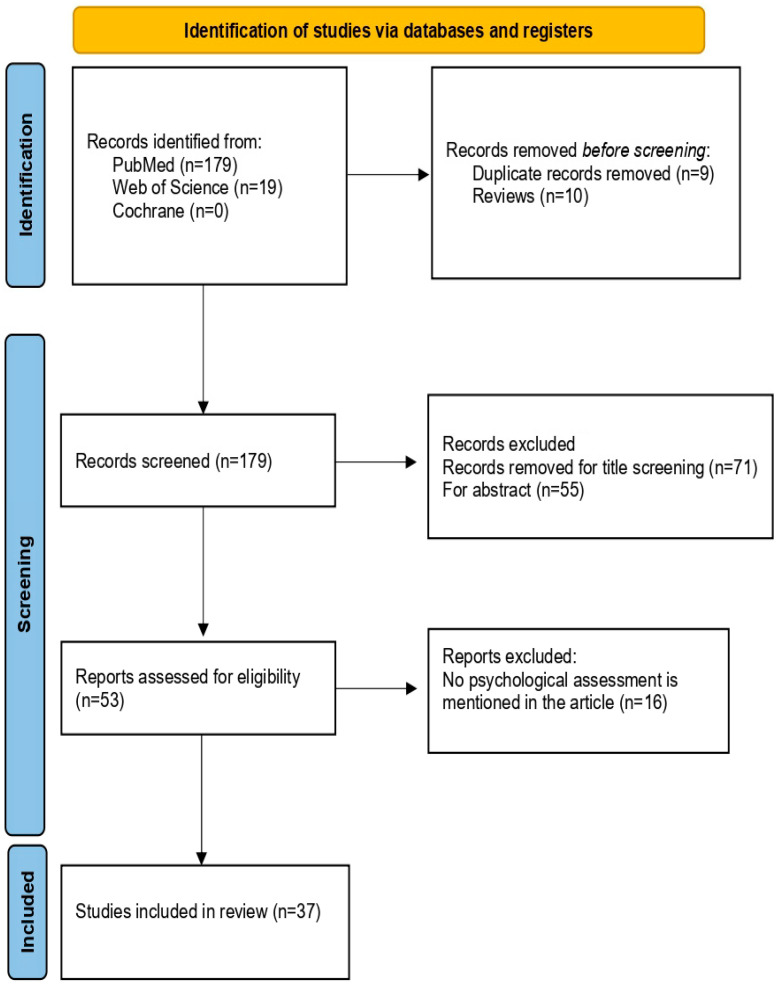
Prisma flow diagram for research strategy.

This systematic review was conducted and reported in accordance with the Preferred Reporting Items for Systematic Review and Meta-Analyses (PRISMA) (see Figure 1).

This review did not have a registration number. All the information was clarified by the corresponding author.

### 2.1. Search Strategy

Articles were selected from research databases—PubMed, Cohrane, and Web of Science—using the following search terms: (“artificial intelligence”[MeSH Terms] OR (“artificial”[All Fields] AND “intelligence”[All Fields]) OR “artificial intelligence”[All Fields]) AND (“machine learning”[MeSH Terms] OR (“machine”[All Fields] AND “learning”[All Fields]) OR “machine learning”[All Fields]) AND (“cognitive dysfunction”[MeSH Terms] OR (“cognitive”[All Fields] AND “dysfunction”[All Fields]) OR “cognitive dysfunction”[All Fields] OR (“mild”[All Fields] AND “cognitive”[All Fields] AND “impairment”[All Fields]) OR “mild cognitive impairment”[All Fields]) AND (“dementia”[MeSH Terms] OR “dementia”[All Fields] OR “dementias”[All Fields] OR “dementia s”[All Fields]) AND ((“cognition”[MeSH Terms] OR “cognition”[All Fields] OR “cognitions”[All Fields] OR “cognitive”[All Fields] OR “cognitively”[All Fields] OR “cognitives”[All Fields]) AND (“assess”[All Fields] OR “assessed”[All Fields] OR “assessement”[All Fields] OR “assesses”[All Fields] OR “assessing”[All Fields] OR “assessment”[All Fields] OR “assessment s”[All Fields] OR “assessments”[All Fields])).

This research was not restricted by the year of publication for the articles considered. However, it is noteworthy that we could not locate any articles predating 2013, given the recency of the topic. Inclusion criteria: (i) articles that enrolled human subjects; (ii) experiments that included cognitive and psychological evaluation in adults; (iii) articles in English language only. Exclusion criteria: (i) reviews and meta-analyses; (ii) duplicated studies.

### 2.2. Study Selection 

A total of 198 articles were identified through database searches in PubMed, Cochrane, and Web of Science. In total, 9 articles duplicated were deleted; 10 reviews were removed; 71 studies were removed for title screening; 55 abstracts were removed; an 16 articles were removed for text screening (Figure 1). In this systematic review, we considered a total of 37 articles about neuropsychological and psychological aspects and assessment, respectively. 

## 3. Results

The present systematic review revealed the various applications of AI and ML algorithms in the diagnosis and psychological assessment of subjects affected by MCI or dementia. A total of 22 articles focused on combining neuropsychological assessment and clinical data for optimizing diagnostic procedures. These studies have employed various neuropsychological tests, along with demographic and clinical data, to diagnose, prevent, or predict cognitive impairment. While some studies have relied solely on neuropsychological and clinical data, others have incorporated biological data, such as magnetic resonance imaging (MRI), to identify risk factors and biomarkers. A total of 10 articles have been conducted with the aim of improving existing test batteries or devising automatic diagnostic tests to facilitate the work of clinicians. Some of these studies also sought to minimize the number of tests required or to select the most suitable set of cognitive tests. The five remaining articles employed virtual reality and games to overcome the limitations of traditional tests. These studies analyze the behavioral data resulting from executive function tasks within Virtual Environments (VEs) to identify biomarkers or distinguish subjects with cognitive impairments from those who are healthy.

## 4. Discussion

This systematic review reveals that the use of AI tools for the prevention of different forms of dementia has garnered significant attention. The early diagnosis and treatment of neurocognitive disorders have become critical healthcare challenges due to the increasing prevalence of dementia and the lack of effective treatments. The growing body of research indicates that AI tools play a crucial role in preventive healthcare strategies. Identifying individuals with cognitive disorders early and understanding the progression of deficits could facilitate timely interventions, which is essential for better outcomes. AI applied to medicine has emerged as a promising solution that can assist clinicians in identifying cognitive deficits in the elderly, providing diagnosis and prognosis. New technologies can help the clinician, as demonstrated by recent studies on the application of AI in the diagnosis of AD and other neurodegenerative diseases. By using data-driven approaches, AI can identify salient patterns that cannot be observed by the human eye or through conventional statistical means. Thus, the integration of AI into clinical practice has the potential to revolutionize the management of neurocognitive disorders, and it is exciting to see what the future holds in this field.

### 4.1. Neuropsychological Assessment and Clinical Data

The majority of the literature examined in this review share a common focus on the employment of AI in combination with demographic and health-related factors, neuropsychological variables, and diverse clinical information in order to develop accurate ML models for optimizing diagnostic procedures, screening for cognitive dysfunction, and predicting cognitive decline. These ML models have been even found to outperform practicing neurologists and neuroradiologists in identifying individuals with MCI or dementia [22,23,24,25]. For instance, a mobile screening test for MCI (mSTS-MCI) was developed due to the limited accuracy of the commonly used Montreal Cognitive Assessment (MoCA) [26]. ML algorithms based on mSTS-MCI and MoCA-K showed higher accuracy compared to original tests when assessing amnestic-MCI (aMCI). Further, various traditional and deep learning models were applied on behavioral data from a facial emotion implicit short-term memory task to classify MCI (MoCA ≤ 25) against standard cognition (MoCA > 25) [27]. The classification achieved reliable accuracy, just below 90% for the best methods. A ML approach was also used to analyze gait data collected from dual-task assessments to detect cognitive impairment [28]. The results of the analysis were compared to the MoCA test. A Support Vector Machine (SVM) was employed to achieve an accuracy rate of 77.17%.

ML models are also useful for screening for cognitive dysfunction, highlighting their potential for use in daily clinical practice [29,30,31]. Among others, a 12 min game-based intelligence test (GBIT) was developed as a reliable and consistent tool for screening for cognitive function in the elderly population [32]. It comprises a 2 min task to assess attention, a 2 min task to evaluate coordination, and a 4 min task to test memory, with a 30 s break in between. Additionally, research has demonstrated that ML algorithms, using neuropsychological, neurophysiological, and clinical data, have the ability to predict progression to MCI [33,34,35,36]. Notably, educational background has been found to be a factor that contributes to cognitive resilience, thus serving as a protective measure against the development of dementia. Other factors that can predict cognitive impairment include age, marital status, and instrumental activities of daily living, as well as baseline MMSE [37], personality traits, and state scores [38]. Additionally, certain Neuropsychiatric Symptoms (NPS) proxies, such as the Neuropsychiatric Inventory Questionnaire (NPI-Q) total severity score, NPI-Q total stress score, and Geriatric Depression Scale (GDS) total score, have been found to predict dementia in MCI [39]. This supports the hypothesis that some NPS can increase the risk of dementia in MCI.

Several studies have incorporated neuroimaging data to predict the progression of MCI to AD and have contributed to a better understanding of the underlying biological mechanisms [40]. For instance, Moradi and colleagues [41] used an aggregate biomarker comprising MRI, age, and cognitive measures to distinguish between progressive and stable MCI patients. Massetti et al. [42] identified the relevant features for predicting the conversion from MCI to AD with an accuracy of 86%, including neuropsychological test scores, MRI data, and Cerebrospinal Fluid (CSF) biomarkers. Notably, an automatically administered and analyzed screening speech-based test was able to predict Amyloid-β (Aβ) positivity and MCI [43]. The test showed an improvement in the detection of MCI by 8.5%, with a significant reduction in false positives of 59.1%. Additionally, the number of positron emission tomography (PET) scans required was reduced by 35.3% and 35.5% for individuals with MCI and cognitively unimpaired individuals, respectively.

### 4.2. Optimizing Neuropsychological Assessment

Despite scoring within the norm of the MMSE, certain subjects may still exhibit cognitive difficulties that are not accounted for by the test. To gain a more comprehensive understanding of a patient’s cognitive state, it is imperative to refine the initial approach to the patient. To this end, researchers have directed their efforts towards leveraging AI to optimize existing test batteries for the identification and classification of cognitive impairment. ML techniques have been implemented to optimize the systematic neuropsychological test battery (NTBs), leading to reduced testing times and improved diagnostic accuracy [44]. Genetic algorithms have also been employed to simplify the testing process by reducing the number of tests required [45]. However, choosing the most appropriate set of cognitive tests for patients is also challenging due to the large number of available options. A study introduced a ML approach for personalized cognitive assessment prioritization, tailoring the assessment selection process to an individual’s brain morphometric characteristics for AD [46]. The method identifies the most effective assessments and prioritizes them at the top of the list. Results from the study on Alzheimer’s Disease Neuroimaging Initiative (ADNI) data showed promising outcomes in identifying patient-specific cognitive biomarkers and assessment tasks based on structural MRI data. Several tests, including Logical Memory (LM), AD Assessment Scale-Cognitive Behavior (ADAS-Cog), Rey Auditory Verbal Learning Test (RAVLT), and the Functional Assessment Questionnaire (FAQ), have been identified as the most reliable predictors for the automatic classification of these pathologies [47] with immediate word recall tasks [48]. Semantic verbal fluency (SVF) tests are regularly used to screen for MCI. In this test, participants are given a category and a time limit to name as many things as possible within that category. Clinicians usually measure task performance manually by counting the number of correct words and errors. Researchers discovered that automatically extracted clusters and switches exhibited a robust correlation (r = 0.9) with manually established values and demonstrated comparable performance in accurately classifying individuals with AD and Related Disorders (ADRD) and MCI from healthy controls (HC) [49]. 

Concerning the Clock Drawing Test (CDT), numerous scoring systems are available, with a predominant reliance on a subjective evaluation by experts. Recent research suggests that clock drawing behavior, obtained digitally and analyzed using ML algorithms, may effectively serve as a screening tool for subtle to mild neuropsychological impairment [50]. One study proposed a computer-aided diagnosis (CAD) system using AI to analyze CDT and provide an automatic diagnosis of cognitive impairment (CI) [51]. The system uses a pre-processing pipeline and a Convolutional Neural Network (CNN) to identify informative patterns for assessing a patient’s cognitive status. The large sample size indicates the high reliability of the proposed method in clinical contexts and demonstrates CAD systems’ suitability in the CDT assessment process.

The local context is also important, as cultural differences may lead to variability in symptom and cognitive manifestations, which could ultimately affect patient treatment and management [52]. The Integrated Cognitive Assessment (ICA) is a 5 min, computerized cognitive assessment tool that uses a rapid categorization task to test information processing speed (IPS) [53]. It detects cognitive impairment using AI and can generalize across populations without the need for population-specific normative data. Unlike other tests, the ICA is not confounded by varying levels of education. It has a comparable diagnostic accuracy to the MoCA and is suitable as a screening test due to its shorter duration and its other advantages over existing tests. Collectively, these studies underscore the promising role of ML in refining neuropsychological assessments.

### 4.3. Virtual Reality and Neuropsychological Assessment

Fully immersive virtual reality (VR) has emerged as a promising tool in the field of neuropsychology. Its potential to overcome the limitations of traditional neuropsychological tests and its suitability for treating executive functions (EFs) within activities of daily living (ADL) make it a significant area of research. While VR was initially used only to assess cognitive states, its high ecological validity, reproducibility of daily life, and the ability to measure behavior responses not available with traditional neuropsychological tests have opened up new avenues for cognitive rehabilitation. In fully immersive VR, supermarkets, kitchens, laboratories, and small squares are usually used as VEs. For instance, the employment of ML models facilitated the analysis of behavioral data derived from executive function tasks conducted within a VR supermarket [54]. Clinical trials revealed 45 potential biomarkers, achieving a 100% accuracy rate in distinguishing individuals with neurocognitive disorders from HC. Subsequently, another ML model was designed to conduct early screening for MCI by analyzing behavioral features from a virtual kiosk test [55]. Notably, patients diagnosed with MCI exhibited indicators such as diminished hand movement speed, reduced proportion of fixation duration, prolonged time required for completion, and an increased number of errors. Additionally, researchers used data obtained from smart home sensors to predict activity quality and assess cognitive health at home [56]. This research revealed a significant correlation between the scores obtained from direct observation and the predicted activity quality.

Panoramix [57] is a powerful tool designed to detect early cognitive markers of AD and MCI. Through the use of advanced ML techniques, it has achieved a remarkable 100% success rate in accurately classifying subjects with cognitive impairments from those who are healthy. The latest version of Panoramix [58] leverages supervised ML techniques to measure predictive cognitive areas with even greater precision. Panoramix 2.0 is an environmentally friendly, non-intrusive, and frustration-free tool that can be helpful for participants with cognitive limitations and can accurately differentiate between cognitive impairments and healthy cognition.

## 5. Conclusions

The present review explored various applications of AI and ML, highlighting how they can significantly contribute to early diagnosis and the optimization of MCI and AD treatment, while reducing the workload for professionals. The studies that combined the principles of neuropsychology with the potential of AI and ML have been examined to identify cognitive markers, develop innovative diagnostic tools, and customize therapies based on the cognitive profiles of patients.

### 5.1. Strengths and Weaknesses

From mobile screening tests, like mSTS-MCI [26], to game-based intelligence tests, such as GBIT [32], and the incorporation of neuroimaging data, these approaches have demonstrated a superior performance compared to traditional methods. This synthesis of established neuropsychological principles with the dynamic capabilities of AI and ML offers a pathway to identify cognitive markers, craft innovative diagnostic tools, and tailor therapies based on the unique cognitive profiles of patients. The exploration of VR and games in neuropsychology further expands the toolkit for cognitive assessment and rehabilitation. The application of ML models in fully immersive VR environments, such as supermarkets [54] and virtual kiosks [55], has shown promise in identifying biomarkers and screening for MCI. These technologies not only redefine the diagnostic landscape but also contribute to a more holistic understanding of cognitive health within real-world contexts. The development of tools like Panoramix demonstrates the potential of environmentally friendly and non-intrusive solutions for detecting early cognitive markers [57,58]. Despite these advancements, there is a need for a shift in focus from technical measures of accuracy to clinical outcomes, risks, and utility. The ethical considerations surrounding AI applications in neuropsychology demand careful examination. As these technologies advance, it becomes imperative to prioritize privacy, obtain informed consent, and scrutinize potential biases in data collection and model predictions. Acknowledging the ethical dimensions ensures that the transformative potential of AI in revolutionizing cognitive healthcare aligns with responsible and patient-centric practices. As part of this research, a key challenge that clinicians face in their day-to-day work was identified. Despite the advent of digital tools and technologies, there is still a notable gap in the market for solutions that genuinely simplify the work of clinicians, without reducing their role to a mere “data entry” function. The analysis suggests that more work is needed in this area, particularly in the development of automated tools that can automatically correct neuropsychological tests. In particular, the potential of a CAD system that leverages AI to analyze the CDT [51] was identified.

While machine learning and AI-driven technologies excel in processing vast amounts of data, they lack the human touch necessary to identify and interpret subtle emotional and behavioral cues critical for accurate patient evaluation. A reliance on algorithms may inadvertently overlook these nuances, potentially impacting the comprehensiveness of the assessment. While AI enhances objectivity and precision, clinicians must navigate a delicate balance between integrating technology without sacrificing the invaluable insights that human connection and context provide in the realm of neuropsychological assessments.

### 5.2. Future Directions

The development of explainable AI is crucial for enhancing healthcare decision making and it is widely anticipated that this field will be explored further in the future. Future studies should aim to bridge the gap between technical performance measures and practical clinical utility, ensuring that AI and ML applications not only provide accurate results but also facilitate the work of clinicians in real-world settings. As the field progresses, the synergy between technological innovation and human expertise will likely redefine the landscape of neuropsychological assessment and contribute significantly to the early detection and management of cognitive disorders. Looking ahead, the successful utilization of AI/ML for predicting the onset of dementia carries the transformative potential to revolutionize healthcare practices globally. Beyond the realms of diagnosis, this integration has the power to mitigate healthcare costs, particularly in the context of aging societies. As AI and ML continue to advance, their thoughtful integration into clinical practice envisions a future where cognitive health is not merely diagnosed early but comprehensively managed, signifying an era of transformative innovation in the field of neuropsychology.

## Data Availability

Not applicable.
